# Analysis of news sentiments using natural language processing and deep learning

**DOI:** 10.1007/s00146-020-01111-x

**Published:** 2020-11-30

**Authors:** Mattia Vicari, Mauro Gaspari

**Affiliations:** 1 University of Bologna, Bologna, Italy; 2 Department of Computer Science and Engineering, University of Bologna, Bologna, Italy

**Keywords:** Deep learning, Machine learning, Natural language processing, Trading signals, Trading, Sentiment analysis, NLP, Trading strategies

## Abstract

This paper investigates if and to what point it is possible to trade on news sentiment and if deep learning (DL), given the current hype on the topic, would be a good tool to do so. DL is built explicitly for dealing with significant amounts of data and performing complex tasks where automatic learning is a necessity. Thanks to its promise to detect complex patterns in a dataset, it may be appealing to those investors that are looking to improve their trading process. Moreover, DL and specifically LSTM seem a good pick from a linguistic perspective too, given its ability to “remember” previous words in a sentence. After having explained how DL models are built, we will use this tool for forecasting the market sentiment using news headlines. The prediction is based on the Dow Jones industrial average by analyzing 25 daily news headlines available between 2008 and 2016, which will then be extended up to 2020. The result will be the indicator used for developing an algorithmic trading strategy. The analysis will be performed on two specific cases that will be pursued over five time-steps and the testing will be developed in real-world scenarios.

## Introduction

Stock forecasting through NLP is at the crossroad between linguistics, machine learning, and behavioral finance (Xing et al. 2018). One of the main NLP techniques applied on financial forecasting is sentiment analysis (Cambria 2016) which concerns the interpretation and classification of emotions within different sources of text data. It is a research area revived in the last decade due to the rise of social media and cheap computing power availability (Brown 2016). Like products and services, market sentiments influence information flow and trading, thus trading firms hope to profit based on forecasts of price trends influenced by sentiments in financial news (Ruiz-Martínez et al. 2012). Is it possible to find predictive power in the stock market’s behavior based on them? It seems to be the case in the work “On the importance of text analysis for stock market prediction” by Lee and MacCartney (2014) that shows, based on text, an improved predictability in the performance of a security. Intuitively, the cause of the stocks’ fluctuation can be the aggregated behavior of the stockholders, who will act based on news (Xing et al. 2018). Although the predicting models reported in the literature have not been able to profit in the long run, many theories and meaningful remarks have been made from the financial markets’ data (Xing et al. 2018). One of the biggest differences between market sentiment problems and linguistics ones is that the ladder has some guarantee of having some type of structures (Perry 2016). There are many models that have been proposed and used in the recent years, each with their positive and negative aspects. Specifically, overly complicated models generally have poor performance, while simpler linear models rely on strong hypotheses, for example, a Gaussian distribution, which does not always apply in real-world cases (Xing et al. 2018). Deep learning seems to be the most fit for this purpose since it has the ability to analyze a great amount of data that NLP needs to understand context and grammatical structures. In this paper, we investigate this scenario, exploring DL for forecasting the market sentiment using news headlines. We assume a basic linguistic framework for preprocessing (stopword, lowercasing, removing or numbers and other special characters), and we reject the common assumption that “positive financial sentiment” = “positive words” and vice versa. The reason is that we do not know if that is the case, and we want the model to learn freely by itself. Taking this assumption into consideration, we are going to test two scenarios, both based on the news published today: case A tries to forecast the movement of the Dow Jones Industrial Average (DJIA) in the next four individual days; case B focuses on time intervals from today to the next 4 days. We discuss the obtained results, and we conclude the paper with a road map for the future.

## Background

### Deep learning

Supervised ML models create mechanisms that can look at examples and produce generalizations (Goldberg 2017). Deep learning (DL)^1^ is a function that imitates the mechanisms of the human brain for finding patterns. Since our case is a binary classification problem (does the DJIA go up or down?), to test our results we used both a binary cross-entropy loss function:

$$- (y\log (p) \, + \, (1 - y)\log (1 - p)).$$

And an accuracy metric:^2^

$$\frac{{{\text{TP}} + {\text{TN}}}}{{{\text{TP}} + {\text{TN}} + {\text{FP}} + {\text{FN}}}}.$$

They are both useful in different ways since the first is used in the training phase, while accuracy is intuitive as long as the classes considered are balanced, like in our case. The basic structure of a neural network (NN) is the neuron: it receives the signal, decides whether to pass the information or not, and sends it to the next neuron. Mathematically, the neuron structure takes some input values × and their relative weights w which are both initialized randomly, and thanks to an “activation function”,^3^ which was the real game-changer (Thanaki 2018), the neurons have the ability to spot non-linear behaviors, that is why they are often addressed as being “Universal Function Approximators”. Since searching over the set of all the possible functions is a difficult task, we need to restrict our scope of action using smaller sets, but by doing so, we added an “inductive bias” (b) that must be taken into account. The most commonly used function for this purpose has the form (Goldberg 2017):

$$NN = f\left( x \right) \, = xW + b$$

$$x \in R^{{d_{{{\text{in}}}} }} ,\quad W \in R^{{d_{{{\text{in}} \times {\text{out}}}} }} ,\quad b \in R^{{d_{{{\text{out}}}} }}$$

The parameters^4^ have the purpose to minimize the loss function over the training set and the validation set (Goldberg 2017). How can NN learn? Through an optimization method that, thanks to a first-order iterative algorithm called gradient descent, can minimize the error function of our model until a local minimum is reached.^5^ Backpropagation is the central mechanism of the learning process which calculates the gradient of the loss function and, by making extensive use of the chain rule, distributes it back through the layers of the NN and adjusts the weights of the neurons for the next iteration. The learning rate used during backpropagation starts with a value of 0.001 and is based on the adaptive momentum estimation (Adam), a popular learning-rate optimization algorithm. Traditionally, the Softmax function is used for giving probability form to the output vector (Thanaki 2018) and that is what we used. We can think of the different neurons as “Lego Bricks” that we can use to create complex architectures (Goldberg 2017). In a feed-forward NN, the workflow is simple since the information only goes…forward (Goldberg 2017). However, when humans read a book, for example, they comprehend every section, sentence or word taking into account what they saw previously (Olah 2015); therefore, a feed-forward NN is not fit for our purposes because it cannot “remember”. Recurrent neural networks (RNN), on the other hand, can catch the sequential nature of the input and can be thought of as multiple copies of the same network, each passing a message to a successor (Olah 2015). A well-known drawback of standard RNN is the vanishing gradients’ problem that can be dramatically reduced using, as we did, a gating-based RNN architecture called long short-term memory^6^ (LSTM).

### NLP and vectorization

Natural language processing (NLP) is a field of artificial intelligence (AI) focused on finding interactions between computers and the human language. Linguistics can be a slippery field for humans, and its intrinsic ambiguity is what makes NLP even more problematic for machines (Millstein 2017) since complexity can appear on many levels: morphological, lexical, syntactic, or semantic. Data preprocessing is a crucial method used to simplify raw text data. In NLP, words are the features^7^ used to find sentiment, based on their frequency in a database (Velay and Daniel 2018). The goal of the language model that is to assign probabilistic sentiment to sentences by trying to capture its context, but to do so, the so-called Markov assumption^8^ is necessary. We use encoding for creating word-embeddings, which are tools used to vectorize words into feature-vectors that a NN can use (Millstein 2017). Word-embeddings are representations of documents where vectors with small distances represent words with closely related meanings. These structures allow us to perform math on texts. With the recent advances in DL, word-embeddings are formed with more accuracy, and they make it easier to compute semantic similarities (Xing et al. 2018). Unfortunately, distributional methods can fall victim of different corpus biases, which can range from cultural to thematic: a common saying is that “Words are similar if used in similar contexts,” but linguistics is more complicated than it looks.^9^ Each model has its pros and cons (Velay and Daniel 2018), the difference stays in the user’s ability to have control over its dataset (Goldberg 2017).

## Learning trading indicators on news

**Fig. 1:**
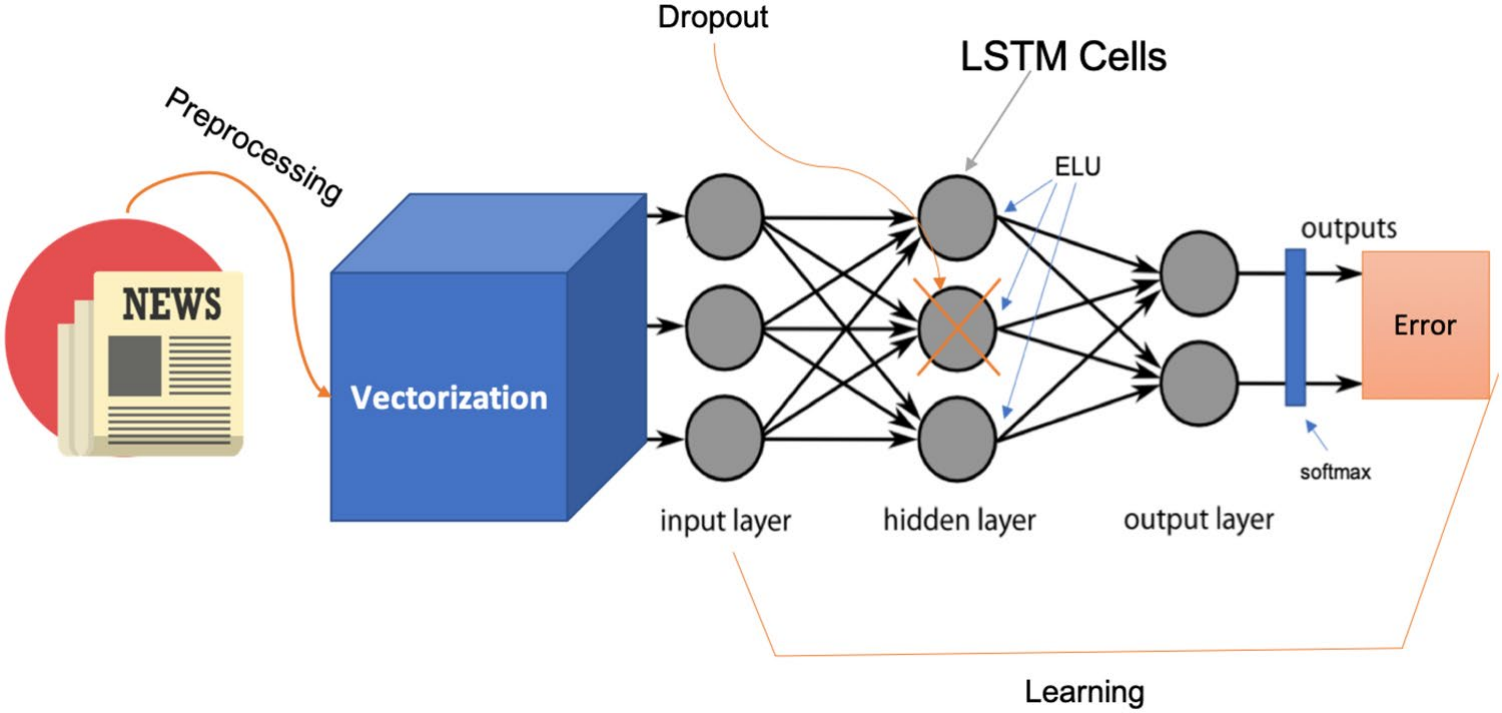
Model structure

A trading indicator is a call for action to buy/sell an asset given a specific condition. When it comes to short-term market behaviors, we are trying to profit on the investors’ “gut-feeling,” but since this phenomenon is something that cannot be unequivocally defined, we must reduce human judgment as much as possible by letting the algorithm learn directly. As shown in Fig. 1, we will start by seeing the chosen dataset. Right after, we will analyze which preprocessing operations have been implemented to ease the computational effort for the model. Then we will see all the components of the DL model put in place and ultimately we will present the results with a real-case scenario.

When working with neural networks, we encounter some limitations that might affect our results (Thanaki 2018): the dimension of the data set, our computing-power availability and the complexity of our model. Just like in the work of Vargas et al. (2017), the dataset is based on news headlines; specifically from the DJIA Database which comes from Kaggle, which contains 25 daily news with economic content from 2008 to 2016 scraped by the author from the most upvoted by the community on Reddit WorldNews: https://www.kaggle.com/aaron7sun/stocknews/home.

Given the fact that the database stretches over almost a decade and contains, for each day considered, a conspicuous amount of news with inherent economic content, we decided that it represents a plausible research instrument. The database was labeled based on whether the DJIA increased or decreased over each time step considered.

## The studied model

**Fig. 2:**
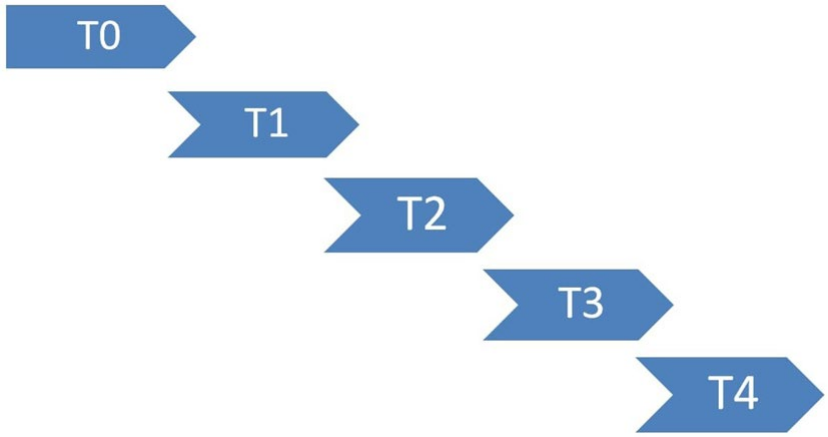
Case A

**Fig. 3:**
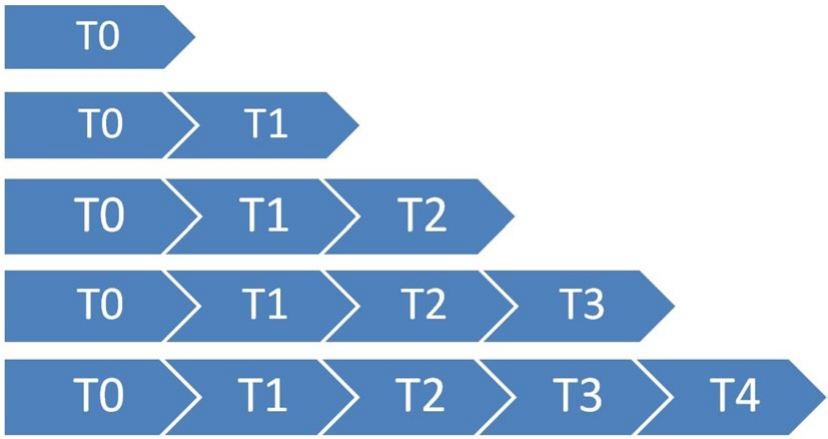
Case B

The focus is on aggregate market indicators and two cases are considered, namely cases A and B as shown, respectively, in Figs. 2 and 3.

The T0 event, common in both instances, analyzes if, based on the news published today, today’s Adjusted closing price is higher than today’s opening price. While, based on the news published today, case A tries to forecast the movement of the DJIA in individual days, case B focuses on time intervals. After defining these market indicators, the preprocessing phase is crucial to reduce the number of independent variables, namely the word tokens, that the algorithms need to learn. At this stage, the news strings need to be merged to represent the general market indicator, from which stopwords, numbers and special elements (e.g. hashtags, etc.) were removed. In addition, every word has been lowercased and only the 3000 most frequent words have been taken into consideration and vectorized into a sequence of numbers thanks to a tokenizer. Furthermore, the labels are transformed into a categorical matrix with as many columns as there are classes, for our case two. The NN^10^ presented in Fig. 1 starts with an embedding layer, which is the input of the model, whose job is to receive the two-dimensional matrix and output a three-dimensional one, which is randomly initialized with a uniform distribution. Then this 3D-matrix is sent to the hidden layer made of LSTM neurons whose weights are randomly initialized following a Glorot Uniform Initialization, which uses an ELU activation function and dropout. Finally, the output layer is composed of two dense neurons and followed by a softmax activation function. Once the model’s structure has been determined, it needs to be appropriately compiled using the ADAM optimizer for backpropagation, which provides a flexible learning rate to the model.

**Fig. 4:**
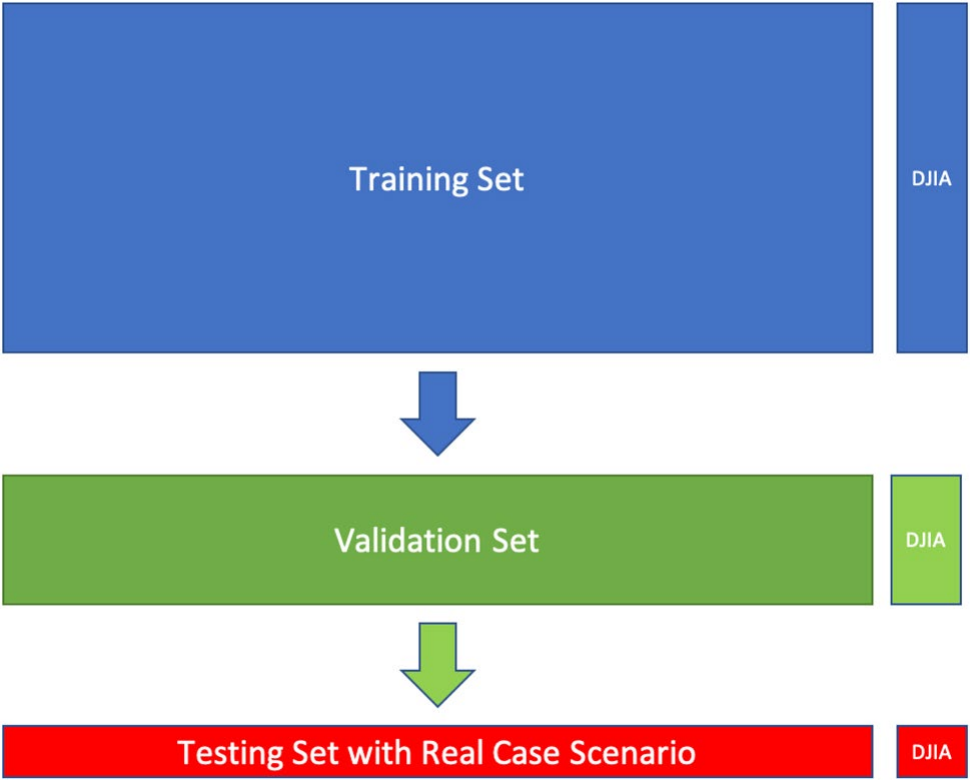
Structure of training, validation and testing sets with DJIA labels

As shown in Fig. 4, the database is then divided into training and validation set with an 80/20 split and evaluated by the binary cross-entropy and accuracy metrics that we previously discussed. Moreover, the training set is split into small pieces called batches (which, for instance, have a dimension of 64 for the T0 case) that are given to the computer one by one for 25 iterations in the training set and two epochs to ease the computational effort when updating the weights.

## Results

Table 1 shows the level of accuracy obtained in this experiment relative to the validation sets:

**Table 1:** Accuracy levels of the validation sets for cases A and B

Time step	Case A (%)	Case B (%)
T0	57.94	57.94
T1	55.85	55.17
T2	54.79	55.59
T3	53.46	55.47
T4	53.60	57.75

Consistent with previous studies (Velay and Daniel 2018), we immediately notice that the accuracy is particularly low since, in both cases, our peaks stick around 58%, which is slightly higher than the flip of a coin.

Besides, in both versions of the model, the highest accuracy appears in the T0 case, behavior that suggests that forecasting attempts within shorter time periods should be preferred, confirming existing literature on the topic (Souma et al. 2019; Sohangir et al. 2018). Nevertheless, we can notice the tendency that the accuracy gradually decreases in case A, while case B shows a less evident decrease that ends with a final increase in T4. Given the low accuracy level, we asked ourselves: How would this model behave in a practical application? Thus, we chose specific news related to major political events that, from our perspective, might have affected the global markets. For this reason, the first testing case looks at major political events that might have caused relevant shifts in the balance of the world:

- Start of Trump’s formal impeachment inquiry.
- Large crowds of protesters gathered in Hong Kong.
- Boris Johnson becomes prime minister.
- Protests for George Floyd’s murder explode.
- COVID-19 was declared a global pandemic by the WHO.

In the second testing category, we decided to look closer at Trump’s presidency. The events chosen were:

- The Summit in Singapore between Trump and Kim Jong-Un.
- Trump signs tariffs on steel and aluminum.
- Trump formally announced US withdrawal from the Paris Agreements.
- Trump signs a big tax cut that was beneficial to big corporations.
- Trump starts the government shutdown to build the wall.

To be consistent with the training and validation set, we manually retrieved from the “News” section of Google 25 news focused on the date of every major event listed above. By comparing each prediction with how the DJIA behaved, we notice that the results are not significant because the results obtained did not show any consistent pattern. Therefore, we agree with Arora et al. (2019) when stating that financial information is extremely unpredictable and that the task of predicting stock movements remains open. For this reason, we thought it could be interesting to keep studying how such a model would behave over longer periods of time by feeding it with more data in the training phase. In fact, for image recognition experiments, DL learning is known for having better performance the more data it sees; therefore, we will follow the same path for financial forecasting. Thus, we decided to extend the original data set until August 2020 and test the same scenario using the same methodology. We retrieved 25 most upvoted daily news from the sub-Reddit/r/worldnews/, just like the author originally did, thanks to the data submissions available in the Pushshift data collections: https://www.files.pushshift.io/reddit/submissions/.

To keep our results comparable, we kept the same NN structure as in the previous case. The results of the experiment using this extended data set in reported in Table 2.

**Table 2:** Accuracy levels of the validation sets for the “extended” datas set: cases A and B

Time step	Case A (%)	Case B (%)
T0	55.02	55.02
T1	53.65	54.01
T2	55.49	50.33
T3	56.43	50.00
T4	54.09	48.83

On the one hand, for the extended case A, the outcome is mixed and there is no added benefit to our initial model. On the extended case B, on the other hand, we notice an even worse forecasting performance. In addition, as in the previous test for individual news, the results obtained did not show any relevant pattern and are not significant. Unlike in the image-recognition field, which is known for improving its performance whenever a more extensive data-set is fed to the deep learning model, for our financial forecasting case we did not obtain the same performance improvement. Why despite increasing the dataset did we get worse results? We analyzed the datasets for the T0 case and the extended T0 case deeper.

The original dataset T0 gives us 2267 trading dates for the DJIA and the DJIA closed higher than the open on 1212 times and closed lower on 1055 days. This means that an algorithm which would guess “DJIA increase” every single day which would result in

- True positives: 1212
- True negatives: 0
- False positives: 1055
- False negatives: 0

At the same time, the extended T0 case shows us that the DJIA went up 1625 days and down 1367 times. Applying again an algorithm which would guess “DJIA increase” every single day would have these characteristics:

- True positives: 1625
- True negatives: 0
- False positives: 1367
- False negatives: 0

Therefore, we would have an accuracy of around 53% for the original T0 case and of around 54% for the extended T0 case, values that are both extremely close to the results we obtained through an optimized DL model. Thus, we presume that the algorithm did not learn anything other than the bias in the data. Further paths worth exploring would be testing DL models with different databases, that could be:

- Using news more focused on a specific security or newspaper.
- Testing cases right after the news comes out.
- Exploiting a dataset with more news per day.
- Exploring a wider variety of NLP methods in the preprocessing phase.

By removing the assumptions “positive financial sentiment” = “positive words”, forecasting sentiment becomes particularly difficult and an elaborated model such as DL the indicator appears weak (Sohangir et al. 2018). In fact, the closer we get into situations where human discretion is involved, the more unstable a model becomes (Goldfarb et al. 2018).

## Discussion and future roadmap

Forecasting consists in grabbing data that we have to generate new information: this is a field that economics has been studying for years, and perhaps by combining the old logic of decision theory and DL methods, we may have a better understanding from a different perspective (Goldfarb et al. 2018). The benefit of DL is that it does not require lots of human effort to effectively provide outputs that were previously considered impossible and a human exclusive. Indeed, AI did not give us more intelligence, but more prediction capacity.

In fact, the innovation of this technology is not the math behind it that existed for decades, but the combination of this knowledge with the Big Data environments that we can access nowadays (Goldfarb et al. 2018). Economics says that by eliminating every obstacle to trading leads to market efficiency. Is it always true? Would the introduction of market agents using trading strategies based on DL and sentiment analysis increase market efficiency? The answer is “it depends”. By introducing a new investing technology we could indeed increase market liquidity and therefore stock markets’ efficiency as a whole. However, the stock market is a complex ecology of interacting players, all with their own strategies and things can go wrong very fast. Let us take a step back and analyze the situation with a few examples from the past that might help us better understand what to expect tomorrow (Buchanan 2012):

- First, the belief that financial innovations such as derivatives could help us reach more market stability and efficiency dominated the last decades (Buchanan 2012). However, those instruments, passed a threshold, profoundly endangered the whole system in 2008 (Buchanan 2012).
- Second, the innovation of High-Frequency Trading (HFT) has improved the stock market as well by reducing trading costs, enhance liquidity, making markets faster and more reactive in calm times, but they do the exact opposite in troubled ones; exactly when the market would need it the most (Buchanan 2012).
- Third, hedge funds typically borrow money to attract investors and increase their profits. However, right before the “Quant meltdown” of August 2007 it was clear the strategies used became too similar, causing their margins to decrease. Therefore, to keep being appealing to investors, managers were slowly forced to increase leverage until it was unsustainable and everything collapsed (Buchanan 2012).

The belief that the world is becoming more efficient and stable than ever thanks to financial innovation (Buchanan 2012) can be misleading. Then why do we always seem surprised when such crises appear? (Buchanan 2012) It is not a coincidence that excessive efficiency can compromise stability (Buchanan 2012). Efficiency means doing more with less, while stability implies the opposite: some extra room to absorb a hit. Given the current hype for AI, these interconnected markets will have access to new ways to invest at incredible speed. In such a context, trading strategies based on sentiment may be particularly prone to the “herd behavior” assuming that these algorithms must train on datasets containing lots of news that may turn out to be extremely similar (Buchanan 2012). In this context, the hypothesis of a “splash crash” ranging across many asset classes does not seem impossible (Buchanan 2012). The global financial crisis has revealed the need to drastically rethink how we regulate the financial system to get ready for when the next recession will strike (Buchanan 2012). Innovation cannot be stopped, but acknowledging its limitations can help us find the best ways to reduce its weaknesses. The fight is rapidly shifting from humans towards machines, but they are playing with real businesses and people, and we must counteract effectively and rapidly in that regard (Buchanan 2012).

## Conclusion

In conclusion, by removing the assumptions “positive financial sentiment” =  “positive words”, financial forecasting becomes particularly difficult and even a DL model does not show better results than the flip of a coin for any of the cases or time-intervals studied. We acknowledge that there are many other paths that could potentially improve our results, but increasing the database size over a longer period of time, common practice in image-recognition problems, does not look like one of them. Despite that, we believe that deep learning methods could potentially be the cause of a relevant danger, due to its proneness towards the “herd behavior”, for the financial system as a whole and thus must be handled carefully.

## Footnotes

1 Also known as a deep neural network or deep neural learning.

2 Where TP means’True Positives’, TN means’True Negatives’, FP means’False Positive’ and FN means’False negatives’.

3 The Activation Function chosen is the Exponential Linear Unit (ELU) since it shows better performance than its Rectified Linear Unit (ReLU) predecessor Hochreiter et al. (2016) by reducing the vanishing gradient problem and by accepting negative values.

4 Where *x* is the input, *W* are the weights, *b* is the inductive bias. Right after are explained the dimensions of the vectors that have been enumerated. These are all cases that we will go over later on but, for example: *x* in our case is a vector whose dimension represents a matrix whose dimensions are represented by the product between the maximum length of the sentences and the random initialization created using the embedding layer for each batch. The notation named ‘*d*_out_’ represents the dimension of the following layer that will receive the output of the current one.

5 For the sake of completeness, it does not tell us the minimum error immediately but shows us the proper direction we need to follow Thanaki (2018).

6 To avoid overfitting of the LSTM structure, we adopted a common regularization method called "Dropout".

7 Features in a model based on AI are individual measurable properties used as input to obtain an output Thanaki (2018); therefore, any attribute can be a feature as long as it is measurable and useful.

8 Which states that the probability of the next word is calculated by looking at the previous one.

9 The clearest example is polysemy.

10 The number of neurons, dropout and epochs named below represent the common T0 case, but have been fine-tuned for each other case using the "babysitting method".
